# Age of Migration and Cognitive Life Expectancies Among Older Latinos: Evidence From the Health and Retirement Study

**DOI:** 10.1093/geroni/igaa057.1904

**Published:** 2020-12-16

**Authors:** Marc Garcia

**Affiliations:** University of Nebraska, Lincoln, Nebraska, United States

## Abstract

This study used data from the Health and Retirement Study (1998-2014) to estimate Sullivan-based life tables of cognitively intact, cognitively impaired/no dementia (CIND), and dementia life expectancies by nativity, age of migration, and sex for older Latinos residing in the United States. Results show foreign-born Latinos, regardless of age of migration or sex, spend a greater number of years after age 50 with CIND compared to U.S.-born Latinos. Furthermore, we document an advantage in total life expectancy and cognitively intact life expectancy among mid-life immigrant men relative to their U.S.-born counterparts. The robust relationship between nativity, age of migration, and cognitive health suggests that the foreign-born may place particularly serious burdens on families and the government. This issue merits special attention in the development of community-based long-term care programs to appropriately target the specific needs of different subgroups of older Latinos who are entering into their last decades of life.

